# „Rheuma (be-)greifen“ – ein multimodales Lehrkonzept zur Verbesserung der rheumatologischen Lehre für Studierende der Humanmedizin

**DOI:** 10.1007/s00393-023-01391-w

**Published:** 2023-07-28

**Authors:** L. Schuster, L. Valor-Méndez, J. Wacker, V. Dannhardt-Thiem, A. Schmidt, J. Knitza, D. Simon, B. Manger, G. Schett, A. Kleyer

**Affiliations:** 1 Friedrich-Alexander-Universität Erlangen-Nürnberg (FAU), Erlangen-Nürnberg, Deutschland; 2 Present Address: Medizinische Klinik 3 – Rheumatologie und Immunologie, Universitätsklinikum Erlangen, Erlangen, Deutschland; 3 Deutsches Zentrum Immuntherapie, Universitätsklinikum Erlangen, Erlangen, Deutschland; 4 Medizinische Fakultät, Friedrich-Alexander-Universität Erlangen-Nürnberg, Erlangen, Deutschland

**Keywords:** Rheumatologie, Medizinische Ausbildung, Virtual Reality, 3‑D-Druck, Rheumality©, Rheumatology, Medical education, Virtual reality, 3D printing, Rheumality©

## Abstract

**Hintergrund:**

Das Bündnis Rheumatologie fordert mithilfe der Kampagne Rheuma2025 eine Verbesserung der studentischen Ausbildung, um in Zukunft die Versorgung rheumatologischer Patientinnen und Patienten zu sichern.

**Fragestellung:**

Wir stellten uns die Frage, ob eine Kombination aus traditionellen und innovativen Lehrmethoden sowohl eine Qualitätsverbesserung der Lehre als auch eine Attraktivitätssteigerung der Fachdisziplin Rheumatologie ermöglicht.

**Material und Methoden:**

Das Lehrkonzept „Rheuma (be-)greifen“, bestehend aus 5 Modulen zu Anamnese mit Schauspielpatientinnen und -patienten, Gelenkultraschall, Gelenkpunktion, 3‑D-Druck von pathologischen Gelenken und Virtual-Reality-Applikation auf Basis realer Fälle in der curricularen Lehre von Studierenden der Humanmedizin, wurde konzipiert und etabliert.

**Ergebnisse:**

Die Evaluation des Lehrkonzeptes bei 93 Studierenden der Humanmedizin erbrachte eine durchgehend hohe Akzeptanz aller Module, welche durchwegs mit „sehr effektiv“ oder „eher effektiv“ bewertet wurden. Module, die unmittelbar auf Patientinnen und Patienten bezogen sind, wie Anamnese mit Schauspielpatientinnen und -patienten, Gelenkultraschall und -punktion, zeigte eine noch etwas höhere Akzeptanz als die Visualisierungsmethoden mittels 3‑D-Druck und VR-Applikation.

**Diskussion:**

Innovative Lehrmethoden können dabei helfen, die Akzeptanz der rheumatologischen Lehre zu stärken, insbesondere wenn klassische Lehrinhalte durch die Verwendung neuartiger Methoden unterstützt werden.

## Abstract (en)

**Background:**

The German Society for Rheumatology, through its campaign Rheuma2025, aims to improve student teaching in order to ensure patient care for rheumatological patients in the future.

**Objective:**

To assess whether a combination of traditional and innovative educational methods provide both an improvement in the quality of teaching and an increase in the attractiveness of rheumatology as a discipline.

**Material and methods:**

Establishment of the teaching concept “*Rheuma (be-)greifen*” consisting of five modules on patient history taking with acting patients, musculoskeletal ultrasound, arthrocentesis, 3D printing of pathological joints and virtual reality applications based on real patient cases in the curricular teaching of medical students.

**Results:**

The evaluation of the teaching concept with 93 students of medicine showed a consistently high acceptance of all modules, which were rated as very effective or rather effective. Direct patient-related modules, such as history taking with acting patients, musculoskeletal ultrasound and arthrocentesis, received even higher acceptance than the visualization methods utilizing 3D printing and virtual reality.

**Conclusion:**

Innovative teaching methods can help to improve the acceptance of teaching in the field of rheumatology, especially when combined with classical teaching contents.

## Hintergrund

Das Versorgungsangebot in der Rheumatologie in Deutschland ist mit 691 an der vertragsärztlichen Versorgung teilnehmenden Ärztinnen und Ärzten nicht ausreichend, um eine adäquate Patientenversorgung anzubieten [4]. Dies resultiert in einer monatelangen Wartezeit für Patientinnen und Patienten, in der häufig bereits irreversible Krankheitsschäden entstehen [3]. Die Deutsche Gesellschaft für Rheumatologie (DGRh) geht dabei in ihrem Memorandum von 2016 von einem Mindestbedarf an 1350 Rheumatologinnen und Rheumatologen aus, um eine bedarfsgerechte Versorgung anbieten zu können [16]. Wie eine Evaluation der DGRh im Jahr 2016 zeigte, ist die studentische Ausbildung im Fach Rheumatologie ebenfalls verbesserungsbedürftig [14]. Aus diesem Grund hat die Kommission Studentische Ausbildung der DGRh seit 2015 gezielte Maßnahmen ergriffen, wie beispielsweise die Erstellung eines Skriptums oder Studierendenprogramme auf dem Jahreskongress der DGRh, um die studentische Lehre zu verbessern. Auch das Bündnis für Rheumatologie arbeitet daran, mithilfe der Kampagne Rheuma2025 die Versorgung für Millionen von Rheumapatientinnen und -patienten in Deutschland zukünftig zu sichern. Kernpfeiler dieser Kampagne ist die Nachwuchsgewinnung über eine verbesserte universitäre Ausbildung und ein fachübergreifendes Systemdenken [12]. Da die Rheumatologie jedoch ein praktisches Fach ist, werden gerade diese Fertigkeiten, darunter Gelenkuntersuchungen durch Palpation oder Gelenkpunktionen, dabei oft nur unzureichend vermittelt. Daher müssen innovative und visuelle Instrumente eingesetzt werden, um als Fach in Konkurrenz zu anderen Fachdisziplinen attraktiv zu bleiben.

## Fragestellung

In unserer Arbeit stellten wir uns die Frage, ob „traditionelle“ Methoden mit neuartigen und innovativen Ansätzen für die Lehre zusammengebracht werden können, mit dem Ziel neben einer Qualitätsverbesserung gleichzeitig die Attraktivität des Fachbereichs Rheumatologie zu steigern.

Um diese Frage beantworten zu können, entwickelten wir ein modulares Lehrkonzept unter dem Begriff *„Rheuma (be-)greifen“*. Es beruht auf der sequenziellen Anwendung verschiedener Untersuchungs- und Visualisierungsmethoden, bestehend aus aktuell 5 klinischen, digitalen und virtuellen Modulen. Grundlage unseres Konzeptes sind dabei neue visuelle Methoden wie der dreidimensionale Druck von arthritisch veränderten Gelenken und Wirbelsäulen reeller Patientinnen und Patienten oder immersive Technologien wie etwa Virtual Reality (VR) oder Augmented Reality (AR). Studierende der Humanmedizin im klinischen Abschnitt, im Rahmen des Rheumatologie Blockpraktikums ab dem 5. Semester, sollen rheumatologische Pathologien nicht nur erkennen, sondern auch nachhaltig verstehen, erleben und begreifen können. Dieses Lehrkonzept ist mittlerweile ein fester Bestandteil der curricularen Lehre des Blockpraktikums Rheumatologie am Universitätsklinikum in Erlangen geworden.

Ein Teil des Blockpraktikums findet vor Ort in unserer Abteilung statt, während ein praktischer Teil in enger Zusammenarbeit mit dem SkillsLab „PERLE“ (*P*raxis *ER*fahren und *LE*rnen) der Medizinischen Fakultät der Friedrich-Alexander-Universität Erlangen-Nürnberg (FAU) durchgeführt wird. Das SkillsLab „PERLE“ ist eine Einrichtung, in der allgemeine Kurse zum Erlernen ärztlicher Fähigkeiten und Fertigkeiten angeboten werden, sodass Studierende praktische Kompetenzen an Modellen und Simulationspatientinnen und -patienten (SPs) erwerben können. Geschulte Tutorinnen und Tutoren führen durch die Module und betreuen die Studierenden im Peer-Teaching.

Dank der jährlichen Förderungen im Rahmen des Innovationsfonds Lehre der FAU^1^, einem internen Förderprogramm, das der qualitativen Verbesserung der Lehre dient, konnten im Jahr 2018 die ersten Teile unseres Lehrkonzeptes umgesetzt und implementiert werden. Außerdem konnten dadurch die notwendige technische Ausstattung sowie die benötigten Modelle bereitgestellt werden.

Das ausgearbeitete multimodale Lehrkonzept besteht aus 5 Modulen mit dem Ziel, praktische Fähigkeiten mit neuen und innovativen Konzepten zu vermitteln. Dabei wurde das System für die VR-Applikation *„Rheumality©“* (Lilly Deutschland GmbH, Bad Homburg v. d. H., Deutschland) fest in den Lehrräumen des SkillsLabs installiert, und die Tutorinnen und Tutoren wurden für die selbstständige Anwendung instruiert. Eine in diesem Zusammenhang veröffentlichte Publikation unserer Arbeitsgruppe zeigte, wie eine erste Version unserer Applikation, die 2‑D- und 3‑D-Daten der pathologischen Gelenke von echten Patientinnen und Patienten mit rheumatoider Arthritis und Psoriasisarthritis in verschiedenen Stadien der Krankheit zum besseren Verständnis der Erkrankungsbilder – nicht nur bei Medizinstudierenden, sondern auch bei Patientinnen und Patienten und sogar bei Rheumatologinnen und Rheumatologen in der Weiterbildung – führt [6, 9]. Auf Grundlage dieses wertvollen Feedbacks wird fortlaufend der Funktionsumfang der Applikation erweitert. Es wurden Prototypen verschiedener Krankheitsbilder, wie z. B. Achsenskelett, Brustwirbelsäule, Becken und periphere Gelenke, mit typischen Pathologien von Patientinnen und Patienten mit axialer und peripherer Spondyloarthritis und rheumatoider Arthritis als 3‑D-gedruckte Modelle produziert [7]. Auf der Basis der erhaltenen Resonanz wurde ein Ultraschallgerät angeschafft und ein entsprechender digitaler Arthrosonographie-Atlas^2^ genutzt, der bereits für ein rheumatologisches Blended-Learning-Wahlpflichtfach für Studierende der Vorklinik etabliert wurde. Zusätzlich wurden Gelenkpunktionsmodelle beschafft und nach entsprechender praktischer Anleitung dem SkillsLab zur Verfügung gestellt. Aufgrund der anhaltenden pandemischen Lage wurde hierfür in einem letzten Schritt noch eine Videoaufzeichnung zur Anleitung der Punktion erstellt.

Ziel unserer Studie war es festzustellen, ob unser Lehrkonzept effektiv zur Wissensvermittlung für Studierende der Humanmedizin in der praktischen rheumatologischen Ausbildung im Rahmen des Blockpraktikums geeignet ist.

## Material und Methoden

**Figure 1:**
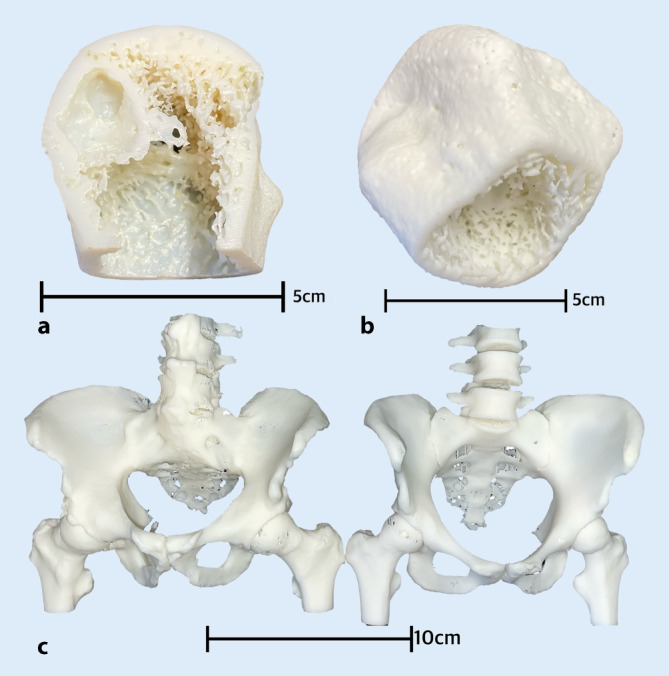

**Figure 2:**
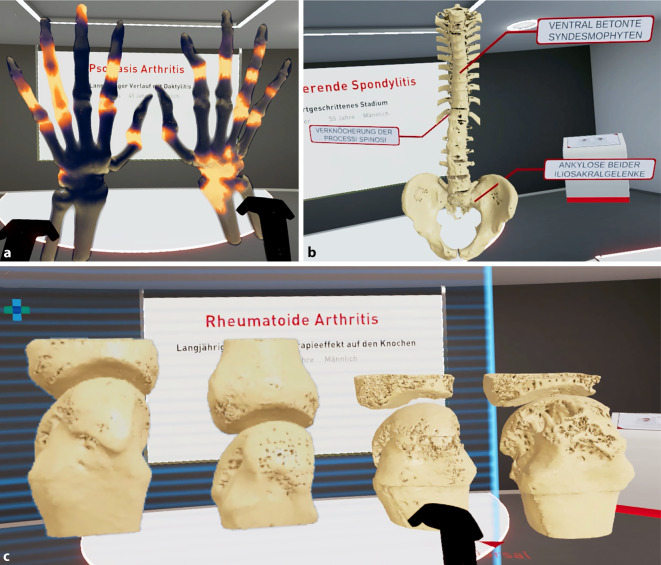

Die Studierenden im Blockpraktikum durchliefen jeweils am zweiten Tag der Lehrwoche die 5 Stationen unseres Lehrmoduls. Dabei wurden sie von Tutorinnen und Tutoren, die vorab von ärztlichem Personal ausführlich für die einzelnen Module geschult wurden, begleitet. Im Anschluss erfolgte eine kontinuierliche Evaluation. Die 5 Module gestalteten sich dabei wie folgt:

- Modul zur Anamneseerhebung mit körperlicher Untersuchung von SPs mit Fokus auf autoimmunen und entzündlichen Erkrankungen: rheumatoide Arthritis, axiale und periphere Spondyloarthritis sowie Psoriasisarthritis. In Zusammenarbeit mit SPs des Simulationspersonenkrankenhaus (SimPatiK) an der FAU wurden Anamneseprotokolle und Untersuchungsabläufe mit dem gezielten Fokus auf rheumatologische Erkrankungsbilder erstellt. In Kleingruppen führten alle Studierenden eine Anamnese mit körperlicher Untersuchung durch. Im Anschluss schätzen die Studierenden ihre eigene Leistung zunächst selbst ein. Auf dieser Grundlage erhielten sie Feedback von ihren Mitstudierenden und von den SPs. Abschließend folgte eine Zusammenfassung und, falls notwendig, eine fachliche Ergänzung durch die Peer-Teacher. Mit dem Einsatz von SPs war es uns möglich, Trainingseinheiten für praktische und kommunikative Kompetenzen als didaktische Methode innerhalb unseres Lehrkonzeptes einzusetzen. Komplettiert wurde dieses Modul durch Vorlesungen während des gesamten Blockpraktikums zu spezifischen Krankheitsprozessen, die bestimmte Organe oder Systeme bei rheumatologisch erkrankten Menschen betreffen.
- Modul Gelenkultraschall: Anatomische Orientierungspunkte für Schulter, Ellenbogen, Knie, Hand und Fingergelenke wurden erklärt und von den Studierenden untereinander aufgesucht. Ein entsprechender digitaler Arthrosonographie-Atlas mit zahlreichen Beispielbildern und Standardschnitten mit ergänzenden Anatomieskizzen zur Orientierung steht dabei zusätzlich zur Verfügung [10].
- Modul Gelenkpunktion: An einem anatomisch genauen Erwachsenen-Kniemodell wurde die Aspiration von Synovialflüssigkeit und Gelenkinjektion sowohl von der lateralen als auch von der medialen Seite unter Palpation geübt. Für Studierende steht über das Videoportal der FAU eine Anleitung zur Kniegelenkpunktion unter sterilen Maßnahmen zur Verfügung.
- Modul 3‑D-Drucke: Anhand von verschiedener Bildgebung, hauptsächlich konventionelle Computertomographie(CT)- und „High resolution peripheral quantitative computed tomography“-Aufnahmen (HR-pQCT) von gesunden, aber auch pathologischen peripheren Gelenken der Wirbelsäule und des Beckens, wurden mittels Stereolitographie-Drucks (SLA) Prototypen angefertigt. Die Drucke wurden in weißem Kunstharz mit einer Schichtdicke von 50 µm hergestellt. Durch anschließendes Reinigen und Aushärten erreichen diese Prototypen eine sehr hohe Formstabilität und eignen sich dadurch gut als Anschauungsobjekt, was wir bereits in weiteren Veröffentlichungen und Kongressbeiträgen beschreiben konnten [8]. Die Abb. 1 zeigt eine Auswahl der verwendeten Modelle für den Einsatz im Blockpraktikum.
- VR-Applikation *Rheumality©:* Die Applikation wurde mit Unterstützung der Firma Lilly Deutschland GmbH sowie mehren universitären Zentren in Deutschland (UK-Erlangen, UK-Jena, UK-Gießen) entwickelt: Mittels einer VR-Brille (HTC Vive, HTC Corporation, Taoyuan, Taiwan) werden die Teilnehmenden durch verschiedene klinische Situationen geführt. Hier werden Daten zur Anamnese, der Vorgeschichte, Diagnose und Bildgebung verschiedener Patientinnen und Patienten immersiv gezeigt. Diese Fallbeispiele sind unabhängig der Therapie ausgewählt, und die Industrie hatte keinen Einfluss auf die Auswahl der Fälle. *Rheumality©* als Instrument ermöglicht eine genaue Bewertung und Visualisierung der strukturellen Schäden der Wirbelsäule, des Beckens und peripherer Gelenke, mit der Möglichkeit, das gezeigte 3‑D-Modell vergrößern und frei um jede Achse drehen zu können. Diese 3‑D-Datensätze wurden auf Basis von hochauflösenden CT-Aufnahmen der peripheren Gelenke, Ganzkörper-PET-CT(Positronenemissionstomographie)-Aufnahmen sowie konventionellen CT-Aufnahmen der Wirbelsäule erstellt. Die Abb. 2 zeigt unterschiedliche computertomographische Aufnahmen, die in der VR-Applikation zu sehen sind.

In dieser Form ist das Blockpraktikum auf einen Einsatz für Medizinstudierende im 9./10. Semester (5./6. klinisches Semester) ausgerichtet. Die Evaluation der Module erfolgte mittels eines Fragebogens unmittelbar nach dem Praktikum und umfasste geschlossene Fragen zur subjektiv empfundenen Wirksamkeit mit der Wissensvermittlung innerhalb der einzelnen Module unter Zuhilfenahme der Likert-Skala mit den Ausprägungen: sehr wirksam, eher wirksam, mäßig wirksam, neutral oder nicht wirksam. Die an diesem Blockpraktikumstag erlernten Fähigkeiten zur Anamnese sowie zu körperlichen und sonographischen Untersuchungen wurden in den darauffolgenden Tagen der Praktikumswoche im Rahmen der ambulanten Sprechstunde und der Stationsvisite anhand realer Patientinnen und Patienten vertieft. Diese Studie wurde von der zuständigen Ethik-Kommission der FAU Erlangen-Nürnberg genehmigt (Votum Nr. 302_20 Bc).

## Ergebnisse

**Figure 3:**
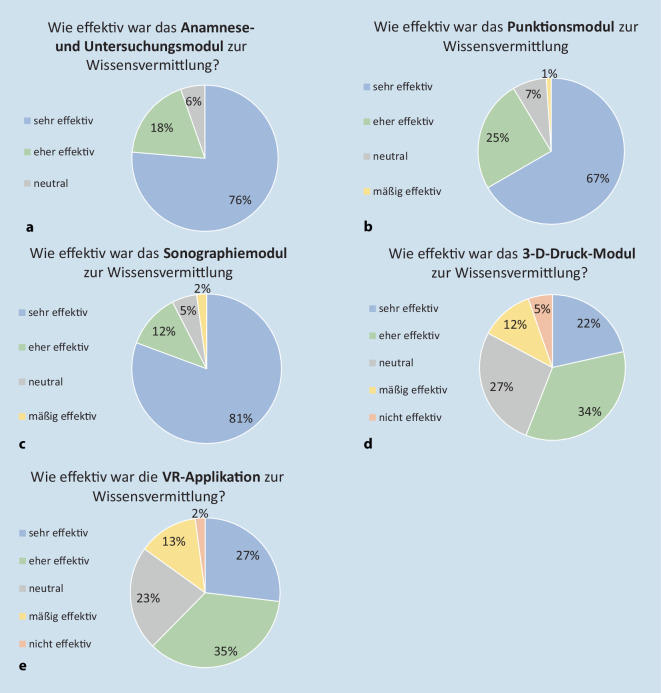

Insgesamt haben 93 Studierende der Humanmedizin im 9. bis 12. Semester (weiblich *n* = 65/männlich *n* = 28/divers *n* = 0) im Altersspektrum von 22 bis 37 Jahren am Lehrkonzept im Rahmen unseres Blockpraktikums teilgenommen. Die Antwortquote betrug 100 % (*n* = 93). Der Großteil der Studierenden bestätigte ein Interesse am Lehrkonzept und bewertete es als sehr effektiv oder eher effektiv. Insbesondere die klinische Anamnese und Untersuchungsmodule mit SPs (76 %, 71/93) sowie die Gelenkpunktion (66 %, 62/93) wurden als sehr effektiv bewertet. Das Ultraschallmodul wurde von 80 % (75/93) der Teilnehmenden ebenfalls als sehr effektiv empfunden. Insgesamt haben 21 % (20/93) der Teilnehmenden die Vermittlung der Pathologien der rheumatoiden Arthritis oder der axialen Spondyloarthritis mittels 3‑D-Modellen als sehr effektiv und 34 % (32/93) als eher effektiv bewertet. Auf die Fragestellung, ob die Visualisierung typischer rheumatologischer Pathologien mit immersiven Technologien wie *Rheumality©* effektiv hinsichtlich des Erkrankungsverständnisses wirksam sei, bewerteten 62 % (58/93) der Befragten dies entweder als effektiv (33) oder als sehr effektiv (25); 2 % (2/93) der Teilnehmenden schätzten diese Form der Wissensvermittlung als nicht effektiv ein. Die Abb. 3 zeigt eine Übersicht über die im Rahmen der Evaluierung erfolgten Befragungsergebnisse.

## Diskussion

Das größte Interesse der befragten Studierenden galt v. a. den Modulen „Anamnese“, „Gelenkpunktion“ und „Ultraschall“, gefolgt von Einheiten zu „*Rheumality©“ *und „3-D-Druck“. Auf der Grundlage dieses Feedbacks haben wir uns noch stärker auf die Verbesserung der Module mit der geringsten Akzeptanz konzentriert.

Neben der Erweiterung der VR-Anwendungen mit mehr Fallbeispielen und der Vorstellung zusätzlicher Bildgebungsverfahren wie PET-CT haben wir den Ansatz verfolgt, unsere Anwendung leichter zugänglich zu machen. Zu diesem Zweck haben wir die VR-Anwendung in eine AR-Anwendung („Rheumality GO!“, Lilly Deutschland GmbH, Bad Homburg v.d.H., Deutschland) überführt, die über digitale Vertriebsplattformen für zahlreiche mobile Endgeräte erhältlich ist^3^. Mit dieser Anwendung können die Auswirkungen von Krankheiten und deren Therapien nun auch ohne kostenintensive Ausrüstung wie VR-Brillen demonstriert werden. Im Durchschnitt ist die Anwendung seit ihrer Veröffentlichung von 67 Nutzerinnen und Nutzern mit Android-Betriebssystemen aktiv installiert und insgesamt 628-mal aus dem Apple App-Store heruntergeladen worden. Die Beurteilungen aus dem App-Store zeigen eine maximale Punktzahl von 5,0 bei insgesamt 7 Bewertungen.

Die Vielfalt an 3‑D-Modellen wurde um Drucke zur Kontrolle des Krankheitsverlaufs erweitert, und es wurden als Pendant zu den Erkrankungen auch Drucke von Aufnahmen vom Normalzustand ohne Hinweis auf das Vorliegen einer rheumatischen Grunderkrankung angefertigt.

Um das Fach Rheumatologie für Medizinstudierende zu optimieren, sind didaktische Lehrkonzepte mit klinischen und virtuellen Elementen in der curricularen rheumatologischen Lehre notwendig. Das praktische Lernen als Lernmethode zur Vermittlung von mehrdimensionalen Kenntnissen ist ein vielversprechender Ansatz für die Entwicklung von klinischem Denken, Kreativität, Kommunikation und Zusammenarbeit bei Studierenden.

Auch ist eine Erweiterung auf zusätzliche Gruppen im klinischen Umfeld denkbar.

In Ergänzung zu der kontinuierlichen Erweiterung der Module erfolgte eine fortlaufende Evaluation des Blockpraktikums mit sehr guten Ergebnissen. Neben den digitalen und praktischen Elementen ist das zusätzlich integrierte Anamnesemodul hervorzuheben, das großen Zuspruch fand. Die Implementierung dieses Systems hat in einer vorläufigen Evaluation im Wintersemester 2020/2021 bereits sehr zufriedenstellende Ergebnisse geliefert [15]. Dieses Modul konnte in der Pandemie digital durchgeführt werden [13].

Der Erwerb von technischen Fähigkeiten und Kenntnissen durch die Simulation führt zu einer Verbesserung der realen klinischen Praxis, was bereits gezeigt werden konnte. Daher wäre es von entscheidender Bedeutung, derartige Simulationen in der Rheumatologie zu fördern und weiterzuentwickeln, beispielsweise durch eine aktive Simulation als Eckpfeiler der Ausbildung von Studierenden der Humanmedizin oder durch technologische Innovationen wie virtuelle und erweiterte Realität, um in einer sicheren Umgebung lernen zu können und ein hohes Maß an Fachwissen erlangen zu können.

Unsere Studie weist Limitationen auf: Durch qualitative Befragungen möchten wir in Zukunft detailliertes Feedback zur Verbesserung sammeln [11]. Zweitens werden durch diese Daten nur die subjektiven Wahrnehmungen der Lernenden widergespiegelt. Eine objektive Bewertung der Wirkung des Simulationskurses auf die Fähigkeiten wurde bislang nicht durchgeführt. Im Vergleich zu Lehrkonzepten anderer Fachdisziplinen könnte unser Lehrmodul durch die Einführung einer Lernerfolgskontrolle mittels Mehrfachauswahlfragen im Nachgang zu den einzelnen Modulen profitieren [5]. Drittens sind SPs kein vollwertiger Ersatz für die Lehre an realen Patientinnen oder Patienten, da gewisse klinische Symptomatiken wie Arthritiden oder Hautveränderungen nicht simuliert werden können. Aus diesem Grund sollte der Einsatz von SPs ergänzend zur echten Praxis im Rahmen der Ausbildung ermöglicht werden. Der Einsatz von SPs kann jedoch bestimmte Aspekte der medizinischen Ausbildung stärker fördern, unter anderem die Schaffung einer standardisierten Lehrumgebung, die auf die einzelnen Ausbildungsphasen ausgerichtet ist [1, 2]. Die Teilnehmenden des Blockpraktikums besuchen in den Tagen nach dem „Rheuma (be-)greifen“-Kurs unsere Ambulanzen. Deshalb betrachten wir das Modul mit SPs als eine gute Übung für Gesprächsführung, Anamnese und Untersuchung.

Die Erweiterung des Konzepts besteht in der Hinzunahme des neuen Spektrums der systemischen Autoimmunerkrankungen (systemischer Lupus erythematodes, Myositiden, Sjögren-Syndrom sowie Vaskulitiden). Die Idee dahinter ist, dass die Studierenden bereits zu Beginn ihrer Ausbildung mit einfachen oder komplexeren Problemen konfrontiert werden, basierend auf klinischen Situationen, denen sie als zukünftige Allgemeinmedizinerinnen und -mediziner begegnen. Ziel ist es, verschiedene Aspekte der rheumatologischen Praxis zu bewerten, wie z. B. die Beziehung zwischen Ärztinnen und Ärzten mit Patientinnen und Patienten, medizinisches Wissen, klinische Fähigkeiten und klinisches Denken, körperliche Untersuchung, ethisches und professionelles Verhalten und Kommunikationsfähigkeiten. Die nur begrenzt zur Verfügung stehenden Ausbildungsstunden in der Rheumatologie für Studierende verlangen, Lehrinhalt hocheffizient zu vermitteln und die Attraktivität für die Fachrichtung Rheumatologie zu steigern.

Teile dieses Lehrkonzeptes konnten bereits an weiteren Universitätsklinika in Jena und Gießen implementiert werden. Ferner können wir uns zur Verfügung stellen, um das Konzept anderen Kliniken zu vermitteln oder dem DGRh-Ausschuss zu präsentieren.

## Fazit

Die vorliegende Arbeit untersuchte die Akzeptanz des von uns entwickelten Ausbildungskonzepts beim Einsatz in der curricularen Lehre von Medizinstudierenden. Die Evaluationsergebnisse zeigen für unseren Ansatz eine hohe Wirksamkeit, auch wenn die eingesetzten innovativen Methoden wie Virtual Reality und 3‑D-Druck nicht an den Erfolg der herkömmlichen praktischen Methoden heranreichen. Zweifellos besteht die Notwendigkeit, unser Programm weiter anzupassen. Zu diesem Zweck werden die Module auch laufend an den eingesetzten Standorten evaluiert. Neben dem Angebot als Online-Fragebogen wurde der Fragenumfang erweitert, um neben der Akzeptanz der Wissensvermittlung das Interesse und die Motivation evaluieren zu können. Die Lehre von Medizinstudierenden im Rahmen des Blockpraktikums leistet einen wichtigen Anteil dazu, eine sichere und patientinnen- und patientenorientierte Gesundheitsversorgung zu gewährleisten. Ob der Einsatz innovativer, interaktiver und kontextbezogener Lehrmethoden den Medizinstudierenden das notwendige Wissen und die Fähigkeiten, Einstellungen und Verhaltensweisen für die zukünftige klinische Praxis umfassend vermittelt, um die klinische Diagnose von muskuloskeletalen Erkrankungen zu verbessern, sollte in zukünftigen Untersuchungen weiter überprüft werden.

## Footnotes

1 https://www.fau.de/education/lehre-an-der-fau/foerderprogramme-fuer-die-lehre/innovationsfonds-lehre/.

2 https://sonographie.org/.

3 https://apps.apple.com/de/app/rheumality-go/id1539520322https://play.google.com/store/apps/details?id=com.lilly.rheumalityAR&gl=DE.
